# Electrophysiological correlates of reward anticipation in subjects with schizophrenia using topographic analysis of variance (TANOVA) – an ERP study

**DOI:** 10.1192/j.eurpsy.2023.812

**Published:** 2023-07-19

**Authors:** A. Perrottelli, T. Koenig, L. Giuliani, P. Pezzella, E. Caporusso, G. M. Giordano, A. Mucci

**Affiliations:** 1 Department of Psychiatry, University of Campania “Luigi Vanvitelli”, Naples, Italy; 2 Translational Research Center, University Hospital of Psychiatry and Psychotherapy, University of Bern, Bern, Switzerland

## Abstract

**Introduction:**

The neurobiological underpinnings of negative symptoms in schizophrenia remain unclear. Previous studies have revealed that in schizophrenia, the anticipatory component of the hedonic experience (anticipatory anhedonia, failure to anticipate reward or pleasurable experiences) is more markedly impaired than the consummatory aspect of pleasure (consummatory anhedonia, in the moment experience of pleasure during pleasurable situations). Several neuroimaging focused on reward prediction deficit have shown dysfunctions in the neuronal circuits that sustain these processes in patients, but findings have not been consistent.

**Objectives:**

The current study aimed at investigating the impairment of reward anticipation in subjects with schizophrenia (SCZ) during the “Monetary Incentive Delay task” (MID task), employing the topographic analysis of event-related potentials (ERPs) with EEG recordings. Furthermore, the associations with negative symptoms and anticipatory and consummatory hedonic experience were investigated.

**Methods:**

EEG data were recorded in thirty SCZ and twenty-three matched HC, during the MID task in which reward and loss cues (incentive cues of positive and negative value) of different magnitude, as well as neutral cues were presented. Anticipation and experience of pleasure were measured by the Temporal Experience of Pleasure Scale (TEPS), while negative symptom dimensions by the Schedule for the Deficit Syndrome (SDS). For the EEG data analysis, the topographic analysis of variance (TANOVA) that uses the global field power of difference maps was used to evaluate between-group differences in scalp topography. Correlation analyses between hedonic experience, negative symptoms and ERPs were performed.

**Results:**

The TANOVA interaction effect (group x cue) was significant in the time window between 140.6 and 195.3 msec after cue presentation (p<.05). Post-hoc analysis showed that significant differences in topography were observed for the reward condition (p=.0006) but not for the loss one (p=.6732) between SCZ and HC. Finally, a significant correlation (p<.01) between t-maps values obtained in the same time-frame and the anticipation of pleasure scores was detected, while no significant correlations were found with the experience of pleasure scores or the severity negative symptom.

**Conclusions:**

SCZ are unable to integrate the incentive magnitude and reward value of future events in the context of their ongoing task. Topographic abnormalities in ERP could be traced already during early stages of reward processing and were associated with anticipation of pleasure, but not with the experience of pleasure or the avolition, suggesting that these constructs might be partially separate.

**Disclosure of Interest:**

None Declared

